# Training to estimate blood glucose and to form associations with initial hunger

**DOI:** 10.1186/1743-7075-3-42

**Published:** 2006-12-08

**Authors:** Mario Ciampolini, Riccardo Bianchi

**Affiliations:** 1 Unit of Preventive Gastroenterology, Department of Pediatrics, University of Florence, Florence, Italy and ONLUS "Nutrizione e Prevenzione", Florence, Italy; 2 Department of Physiology and Pharmacology, State University of New York Downstate Medical Center, Brooklyn, New York, USA

## Abstract

**Background:**

The will to eat is a decision associated with conditioned responses and with unconditioned body sensations that reflect changes in metabolic biomarkers. Here, we investigate whether this decision can be delayed until blood glucose is allowed to fall to low levels, when presumably feeding behavior is mostly unconditioned. Following such an eating pattern might avoid some of the metabolic risk factors that are associated with high glycemia.

**Results:**

In this 7-week study, patients were trained to estimate their blood glucose at meal times by associating feelings of hunger with glycemic levels determined by standard blood glucose monitors and to eat only when glycemia was < 85 mg/dL. At the end of the 7-week training period, estimated and measured glycemic values were found to be linearly correlated in the trained group (r = 0.82; p = 0.0001) but not in the control (untrained) group (r = 0.10; p = 0.40). Fewer subjects in the trained group were hungry than those in the control group (p = 0.001). The 18 hungry subjects of the trained group had significantly lower glucose levels (80.1 ± 6.3 mg/dL) than the 42 hungry control subjects (89.2 ± 10.2 mg/dL; p = 0.01). Moreover, the trained hungry subjects estimated their glycemia (78.1 ± 6.7 mg/dL; estimation error: 3.2 ± 2.4% of the measured glycemia) more accurately than the control hungry subjects (75.9 ± 9.8 mg/dL; estimation error: 16.7 ± 11.0%; p = 0.0001). Also the estimation error of the entire trained group (4.7 ± 3.6%) was significantly lower than that of the control group (17.1 ± 11.5%; p = 0.0001). A value of glycemia at initial feelings of hunger was provisionally identified as 87 mg/dL. Below this level, estimation showed lower error in both trained (p = 0.04) and control subjects (p = 0.001).

**Conclusion:**

Subjects could be trained to accurately estimate their blood glucose and to recognize their sensations of initial hunger at low glucose concentrations. These results suggest that it is possible to make a behavioral distinction between unconditioned and conditioned hunger, and to achieve a cognitive will to eat by training.

## Background

The will to eat is a decision associated with conditioned responses and with body feelings reflecting changes in metabolic biomarkers. The body feelings are often described as hunger, but have components that are strongly conditioned by time, social, and metabolic factors, for which there are salient unconditioned physiologic correlates. Blood glucose has long been considered a biomarker of hunger [1]. In extensive rat studies, Steffens [2] measured glucose at discrete intervals, and showed that blood glucose concentration declined before a meal, remained at a lower plateau until a meal started, and rose sharply shortly after the initiation of a meal. Transient blood glucose declines coincided with spontaneous feelings of hunger and meal initiation in humans and rats, suggesting that these feelings correlate with metabolic insufficiency [3-6]. This condition of hunger was associated with glucose concentrations of 80 mg/dL or lower in humans [1,3-6] and was exacerbated by injection or infusion of insulin [7].

Blood glucose has a central role in the regulation of energy metabolism. It provides energy to the brain, has limited and exhaustible storage, is regulated by the availability of other fuels, and its blood levels correlate with the time interval between spontaneously requested meals [3,8-10]. Our previous investigations indicated that food request in infants [11,12] and diary reports of hunger in adequately trained children and adults [13] were associated with significantly lower glycemic concentrations than conditioned responses were before any training, and that these levels were lower than 85 mg/dL after training [11-13]. Hunger at comparable low glucose concentrations has been reported in time-blinded subjects [3-6]. In the present investigation, we test if appropriate training can lead to recognition of initial hunger at glycemia below 85 mg/dL. We hypothesize that feelings of hunger or discomfort might provide an indicator of the adequacy of glycemia and energy state. Eating in response to these lower blood glucose concentrations rather than to conditioned signals may improve energy balance and, in addition, reduce metabolic risk factors [8-10].

Previous investigations have reported the use of hunger feelings with [11,13] or without [12,14-16] metabolic biomarkers to allow intake and control of energy balance. The current study investigated the associations of subjective estimation, consummatory behavior, and glycemia in trained subjects versus control subjects at breakfast-time to evaluate the subjective feelings of hunger as meal-start signals and to distinguish whether they were either unconditioned or conditioned after training.

The investigation was carried out in patients with functional disorders of the bowel such as dyspepsia, abdominal pain, and diarrhea [17]. Data from this group of patient provide the basis for studies on the effects of behavioral control of feeding on intestinal diseases in adults, as it has been obtained in infants [11,14] and children [12].

## Methods

### Setting

**Figure 1:**
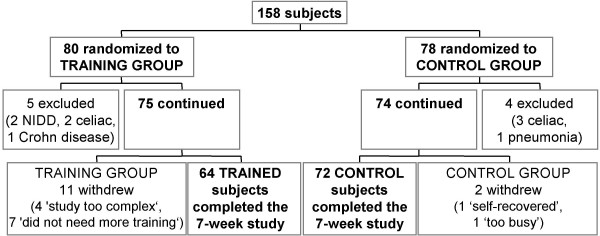
**Consort flow chart**. Randomization of the subjects recruited for this study into trained and control (untrained) groups. The subjects were men and women, 18 to 60 years of age, with recurrent functional disorders of diarrhea, abdominal pain, or dyspepsia.

**Figure 2:**
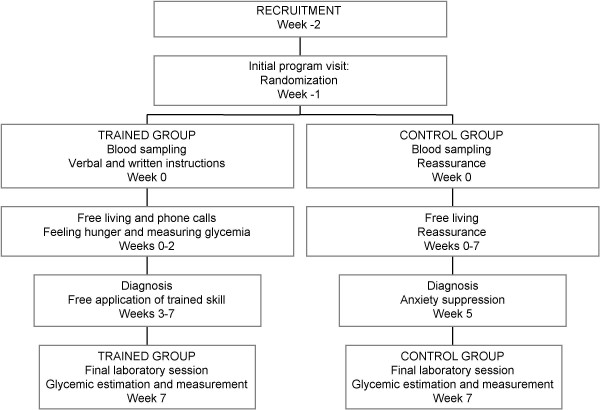
**Investigation design**. A randomized and controlled 7-week pilot clinical investigation to study the acquisition of the capacity to estimate blood glucose by body feelings after adequate training.

In this 7-week pilot study, 158 adults suffering from diarrhea, abdominal pain, and dyspepsia were recruited and randomized to experimental (trained; n = 80) and control (untrained; n = 78) groups (Figure 1). Informed consent was obtained at the initial meeting from all the participants, and the local Hospital Committee approved the study according to the Helsinki Declaration. The subjects did not have impaired glucose tolerance or morphological, physical, or biochemical signs of diseases. Reactive C protein was normal. All subjects reduced work for 3–4 days at the beginning of the experiment and then conducted their normal routine. The experimental group was trained with tutorial assistance while the control group followed their normal routine (Figure 2). After 7 weeks, 64 trained and 72 control subjects completed the program (Figure 1; Table 1). In the final investigative session (week 7; Figure 2), they were asked to estimate their glucose concentrations in the laboratory and these values were compared to those determined through a glucose autoanalyzer. Glycemic measurements were reported on seven-day food diaries that were available before training and in the 7^th ^experimental week.

**Table 1:** Trained and control (untrained) groups at baseline and after seven weeks at the final investigative session.

	**Trained group**		**Control group**	
	**Baseline**	**Investigative**	**Baseline**	**Investigative**

Weeks after baseline	0	7	0	7
Number of subjects	64	64	72	72
Age (years)	37.2 ± 11.0^*1*^	37.4 ± 11.1	37.7 ± 10.6	37.9 ± 10.7
Gender (F/M)	38/26	38/26	46/26	46/26
Overweight/normal-weight^*2*^	22/42	20/44	20/52	20/52
Weight (kg)	68.4 ± 15.7	66.2 ± 14.6^*3*^	63.9 ± 10.6	63.2 ± 10.7
Body Mass Index (BMI)	24.0 ± 4.7	23.6 ± 4.6^*3*^	22.8 ± 2.7	22.6 ± 2.8

^*1 *^Mean ± SD.

^*2 *^BMI = weight (kg)/height^2 ^(m^2^); Overweight: BMI > 25; Normal-weight: BMI < 25.

^*3 *^Not significant *vs *baseline.

### Measurement of glycemia and validation

Subjects in training measured capillary blood by glucometer (a portable potentiometer for whole blood glucose measurement: Glucocard Memory; Menarini Diagnostics; Florence, Italy) in the quarter-of-an-hour before intended meal consumption. Accuracy of measurements by the glucometer was validated at periodic laboratory visits with measurements by autoanalyzer on blood samples from the same subjects. In contrast, control subjects did not have their glycemia measured until the final laboratory session.

### Estimation of glycemia and intake adjustment

On the first training day, subjects were told to ignore previous meal times and to pay attention to their feelings of hunger or discomfort. At the earliest feelings of hunger or discomfort, the subjects measured glucose concentrations with the portable instrument. This first event of hunger appeared after the training session with a time interval that varied widely from 0 up to 48 hours (average 2 h) and was often far from the usual meal times during the next 3 training days. This suggests that the recorded behavioral responses were largely spontaneous (unconditioned). Measurements obtained less than 1 h after a small amount of food consumption, intense physical activity, or changes in environmental temperature were excluded from the analysis. When glycemia was higher than 85 mg/dL, patients were instructed to delay or skip the meal, to engage in some activity as a distraction from food, and to wait for the spontaneous development of novel hunger feelings for at least one hour before making further blood glucose measurements. When glycemia was under 85 mg/dL, patients were instructed to remember their feelings and to proceed to meal consumption. The glycemic level of 85 mg/dL was chosen based on the hypothesis, suggested by previous studies, that it represents the upper limit of homeostatic control of feeding [3-6,11-14]. The subjects in training attempted to identify the initial hunger or discomfort that was in reliable (± 4 mg/dL) association with a particular blood glucose level below 85 mg/dL. During the first 3–4 days, energy intake was decreased and the amount of fruits and vegetables was increased (0.5–1 kg per day) to reduce conditioned feeding behavior and to promote early occurrence of spontaneous events of hunger outside of the usual meal time. Following meals with low glycemic index [18], hunger events could be detected and sustained for at least 1 h without substantial impairment of daily activity. Subjects were instructed to start a meal within 1 h of the appearance of these hunger events. They were prohibited from sustaining hunger for longer than 1 h, to avoid glycemic declines below 65 mg/dL that are known to induce counter-regulatory glucose responses [19]. The subjects repeated and refined this procedure three times a day for at least two weeks. Phone assistance was provided for the subject to describe the events of hunger and to report the times of occurrence, glycemic values, food energy-content, energy expenditure factors, and meal composition adjustments. After this training period, patients annotated their estimations of glycemia before the measurements.

### Final session

At the final investigative session, the subjects returned to the laboratory, stated whether they were hungry or not hungry, and estimated their glucose concentrations before blood sampling and before breakfast. Control subjects had ignored the relation between glycemia (referred to the subjects as "nutrient levels") and feelings of hunger up to the final session. They were asked to estimate their glucose levels referring to a range of values that could vary between the extremes of 60 mg/dL during intense hunger and 110 mg/dL after a satiating meal. Blood was sampled, centrifuged immediately, and analyzed in duplicate. The subjects were then free to eat food that they brought from home or from the hospital cafeteria under the observation of an investigator.

### Statistics

Values were expressed as means ± SD. The analyses included the *t*-test on difference and analyses of simple, linear correlation (r = correlation coefficient in linear regression), agreement limits and estimation error between the estimated and measured values of glucose. The estimation of error was calculated as the mean ± SD of the absolute values of differences from the reference measurement. The significance of difference and correlation was analyzed by two-tailed *t*-test analysis and Yates test, and was set at p < 0.05 when one difference was analyzed between two groups and at p < 0.025 when two differences were analyzed between the same two subject groups, e.g. measured glycemia and estimation error (Table 2) [20]. Excel 5 (Microsoft Corporation, Redmond, WA, USA) was used for the analyses.

**Table 2:** Estimated *versus *measured blood glucose at the final laboratory session (week 7).

	**N**	**Estimated blood glucose**	**Measured blood glucose**	**Difference (Estimated - Measured)**	**Estimation error (%)**
All Trained	64	84.9 ± 7.8^*1*^	87.2 ± 7.9^*2*^	-2.3 ± 4.7^*3*^	4.1 ± 3.1 (4.7 ± 3.6)^*4*^
Hungry Trained^*5*^	18	78.1 ± 6.7	80.1 ± 6.3	-2.0 ± 2.5^*3*^	2.6 ± 1.9 (3.2 ± 2.4)
Not-hungry Trained^*6*^	46	87.6 ± 6.5	90.0 ± 6.6^*7*^	-2.4 ± 5.3^*3*^	4.8 ± 3.2 (5.4 ± 3.6)
					
All Controls	72	78.5 ± 11.6	89.8 ± 10.5^*8*^	-11.3 ± 14.8^*9,10*^	15.4 ± 10.4 (17.1 ± 11.5)
Hungry Controls^*5*^	42^*11*^	75.9 ± 9.8	89.2 ± 10.2^*7*^	-13.3 ± 11.9^*12,13*^	14.9 ± 9.8 (16.7 ± 11.0)
Not-hungry Controls^*6*^	30	82.2 ± 12.9	90.6 ± 10.9	-8.4 ± 17.9^*14,15*^	16.1 ± 11.3 (17.8 ± 12.4)

^*1 *^Mean ± SD, mg/dL. Subjects stated to be either hungry or not hungry and they estimated their blood glucose at the hospital laboratory before breakfast.

^*2 *^Measurements performed by hospital autoanalyzer.

^*3 *^Estimated less measured blood glucose, significant at p < 0.01.

^*4 *^Absolute value of difference between estimated and measured blood glucose and, inside parenthesis, % of measurement.

^*5 *^Subjects who declared feeling hungry at the laboratory investigative session. The agreement limits (mean difference ± 2SD) were -7.0 to +3.0 mg/dL and -41.3 to +18.6 mg/dL in trained and control groups, respectively.

^*6 *^Subjects reporting to be not hungry at the laboratory investigative session. The agreement limits were -12.9 to +8.2 mg/dL and -45.0 to +28.0 mg/dL in trained and control groups, respectively.

^*7 *^p < 0.01 *vs *trained hungry subjects in the respective column.

^*8 *^p = 0.08, not significant, *vs *all 64 trained subjects.

^*9 *^F = 10.6, p = 0.0001 on the difference between estimated and measured blood glucose.

^*10 *^*t*-test p = 0.0001 *vs *all trained subjects.

^*11 *^p = 0.001 *vs *number of hungry subjects in the trained group.

^*12 *^F = 24.6, p = 0.0001 on the difference between estimated and measured blood glucose.

^*13 *^*t *test p = 0.0001 *vs *trained hungry subjects.

^*14 *^F = 11.9, p = 0.0001 on the difference between estimated and measured blood glucose.

^*15 *^*t *test p = 0.07 *vs *trained not-hungry subjects.

## Results

Sixty-four subjects were trained to regulate eating at home by measuring blood glucose during feelings of hunger. The association between feelings and glucose readings were reported by phone, and could be evaluated during the 7 weeks of training (see Methods). Subjects showed an estimation error lower than ± 4 mg/dL after less than a week of training (n = 8) or within the first two weeks of training (n = 47). The remaining 9 subjects either reached an estimation error lower than ± 4 mg/dL in > 2 weeks or still showed an estimation error higher than ± 4 mg/dL at the end of the 7-week-training.

### Hungry subjects (gastric hunger)

**Figure 3:**
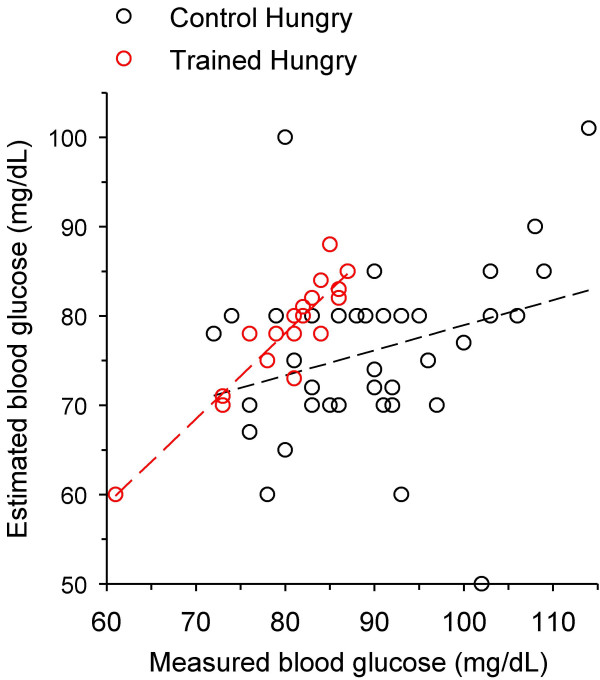
**Estimated *vs *measured blood glucose of subjects reporting to be hungry at the final laboratory investigative session**. *Hollow red circles*, trained hungry subjects (n = 18); *hollow black circles*, control (untrained) hungry subjects (n = 42). Linear correlation was significant for the trained data (*dashed red line*; r = 0.92; p = 0.0001) but not for the control data (*dashed black line*; r = 0.29, p = 0.06).

At the final session, the number of trained subjects stating that they were hungry (18 of 64) was significantly lower than that of hungry control subjects (42 out of 72; Table 2). All hungry subjects described the hunger feeling as gastric emptiness or gastric pangs. In the hungry trained group, the mean estimated glycemic concentration was 78.1 ± 6.7 and the mean measured value was 80.1 ± 6.3 mg/dL (Table 2; Figure 3). This measured glycemia was significantly lower than the measurements in hungry control subjects (89.2 ± 10.2 mg/dL) and in not-hungry subjects of both trained (90.0 ± 6.6 mg/dL) and control (90.6 ± 10.9 mg/dL) groups (Table 2).

The estimation error (the absolute value of the difference between estimated and measured glucose) in the hungry trained group (2.6 ± 1.9 mg/dL; 3.2 ± 2.4% of the measured value) was significantly lower than that in the hungry control group (14.9 ± 9.8 mg/dL; 16.7 ± 11.0%; Table 2; Figure 3). Linear regressions of the values in the hungry groups in Figure 3 also show that there was significant correlation between estimated and measured glycemia in the trained group (r = 0.92; p = 0.0001) but not in the control group (r = 0.29; p = 0.06).

### Not-hungry subjects (hunger equivalents)

**Figure 4:**
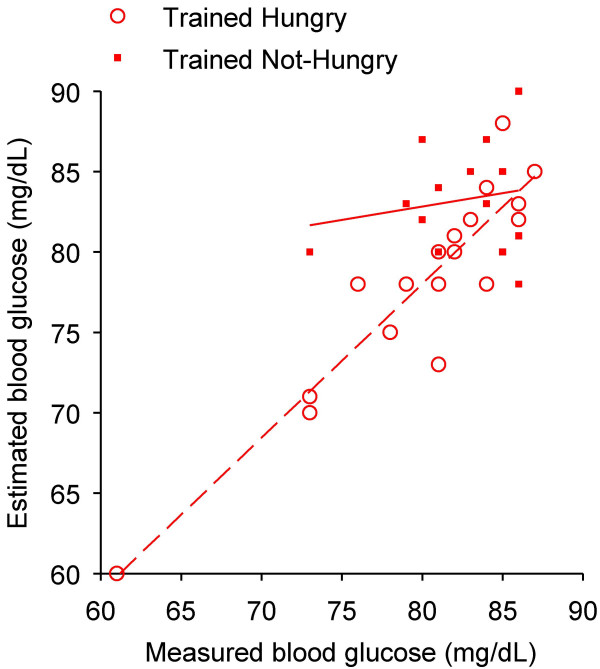
**Estimated *vs *measured blood glucose of trained subjects with levels below 87 mg/dL at the final session**. The highest glycemic value measured in trained hungry subjects was 87 mg/dL. Below this value of measured blood glucose, 18 subjects reported to be hungry (*hollow red circles*) and 14 subjects were not hungry (*filled red squares*). Linear regression is significant for the hungry subjects (*dashed red line*; r = 0.92; p = 0.0001) but not for those not hungry (*solid red line*; r = 0.18; p = 0.54).

**Figure 5:**
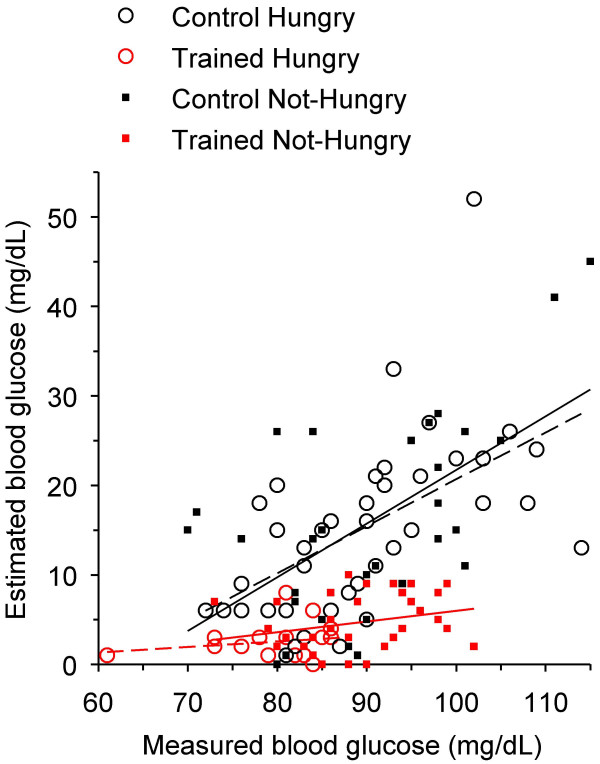
**Estimation error *vs *measured blood glucose in the trained and control groups**. Consistent with previous figures, symbols and regression lines are: *hollow red circles *and *dashed red line*, trained hungry subjects (n = 18; r = 0.20; p = 0.43); *filled red squares *and *solid red line*, trained not-hungry subjects (n = 46; r = 0.24; p = 0.18); *hollow black circles *and *dashed black line*, control hungry subjects (n = 42; r = 0.55; p = 0.0001); *filled black squares *and *solid black line*, control not-hungry subjects (n = 30; r = 0.58; p = 0.001).

The trained and control subjects that were not hungry at the final investigative session significantly underestimated their glucose levels. The estimation errors were 4.8 ± 3.2 mg/dL and 16.1 ± 11.3 mg/dL in trained and control groups, respectively (Table 2). The linear correlation between estimated and measured glycemia was highly significant (r = 0.68; p = 0.0001) in the trained group and not significant in controls (r = -0.12; p = 0.32). The difference between trained and control groups did not depend on gender, age, number of years at school, weight, or body mass index (Table 1). Fourteen out of 46 trained subjects who were not hungry had glucose concentrations below 87 mg/dL, the maximum limit of glycemia of those who were hungry (Figure 4). These 14 subjects showed an average estimation error of 4.5 ± 3.1% of the measured glycemia, which did not significantly differ from the estimation error of the 18 trained subjects who were hungry (3.2 ± 2.4%; p = 0.20). Under 87 mg/dL, estimation error was low in both trained and control groups (n = 32; 3.8 ± 3.7% and n = 31; 13.5 ± 8.9% of the measurement, respectively), independently of the subject's statement on hunger. In subjects with values above 87 mg/dL of glycemia, the estimation error increased significantly to 5.7 ± 3.7% (trained; n = 32; p = 0.04; Figure 5) and to 19.5 ± 11.8% (controls; n = 41; p = 0.001).

Despite their not being hungry, 12 of 14 trained subjects under 87 mg/dL and 3 of 32 above 87 mg/dL (p = 0.001) described the subtle feelings they employed to estimate glycemic concentrations. Thus, compared to controls – who did not report equivalents of hunger (n = 30) – a significantly higher proportion of the 46 not-hungry trained subject (p = 0.001) was able to report feelings other than gastric hunger, which were useful in estimating their glycemic levels, and this ability prevailed below 87 mg/dL. In their reports, these 15 subjects described physical (3 subjects) or mental (10) weakness or abdominal changes in tension or movement (2). Another 6 of the 46 not-hungry trained subjects, but none of the control subjects, had felt gastric hunger before entering the hospital for the final session; however the feeling faded while waiting for the laboratory session.

In the not-hungry subjects' reports, the feelings of mental weakness consisted of difficulty in sustained mental concentration, impatience, irritability, drowsiness, gnawing feeling, loss of enthusiasm and effectiveness at mental work, or poor mood at their jobs. The mental feelings emerged alone or in addition to gastric or other feelings and ceased with the meal. Sensing impairment during physical activity was associated with heavy physical exercise outdoors and often accompanied a change from a sedentary life style. This feeling was used regularly to indicate meal signal with an increased requirement of high-energy-dense food for the next meal(s). The prevalence of these 'hunger equivalents' ranged from an occasional occurrence to less than 15% of the meals in the phone reports. Two subjects reported that they never felt (gastric) hunger, but estimated glycemic concentrations within 6% estimation error always by assessing mental or muscular weakness during training or during the final investigative session. In their reports, these subjects consumed meals at glycemic estimation of 78 to 85 mg/dL.

### Cognitive adaptation to the glycemic concentrations at initial feelings of hunger

At the final laboratory session, the 64 trained subjects showed a decrease of 43.1% in reporting hunger events before breakfast compared to the reported events of the previous week (Table 3). In contrast, the corresponding decrease in hunger reports of the 72 controls was only 11.7% (p < 0.0001; Table 3). Compared to the diary reports of the last training week, the 64 trained subjects also decreased breakfast consumption by 13.7%, significantly more than control subjects (3.8% decrease; p < 0.01; Table 3). The significant reduction in prevalence of attaining the feelings of initial hunger and consuming breakfast at the final session in trained subjects suggests maintenance of surveillance of body feelings and adaptation of intake to this indicator.

**Table 3:** Number of hunger events and breakfast consumptions during the 7^th ^week of training (diary) and at the final laboratory session in trained (n = 64) and control (n = 72) subjects.

	**Trained group**	**Control group**
Hunger events in diary^*1*^	71.2% (319/448)	70.0% (353/504)
Hunger events at the final session	28.1% (18/64)	58.3% (42/72)
Difference in hunger reports (% final - % diary)	-43.1%	-11.7%^*2*^
Breakfast consumptions in diary	74.6% (334/448)	85.7% (432/504)
Breakfast consumptions at the final session	60.9% (39/64)	81.9% (59/72)
Difference in breakfast consumption (% final - % diary)	-13.7%	-3.8%^*3*^

^*1 *^Data are reported as percentages of the total (n values are indicated in parenthesis).

^*2 *^(p < 0.0001) *vs *decrease in trained subjects (Chi square).

^*3 *^(p < 0.01) *vs *decrease in trained subjects (Chi square).

## Discussion

The main result of this study is that adult individuals can be trained to accurately estimate their glucose levels at meal times. This cognition was achieved by conditioning the subjects to associate feelings of hunger with low glucose concentrations (Figure 5, *red symbols*). In contrast, control (untrained) subjects were unable to recognize their glycemic levels at meal times (Figure 5, *black symbols*) and expressed the will to eat at a wide range of glycemic values.

These findings suggest (1) that food consumption at high glycemic concentrations in control subjects may lead to higher energy intake than in trained subjects [8-14], and (2) that the lack of correlation between food consumption and glycemia may, at least in part, explain why part of the population cannot maintain its energy balance. Since our study was conducted on subjects with gastro-enteric disorders, it remains to be determined whether such deficit of association between food intake and glycemia is limited to this patient population or is a more general mechanism involved in other metabolic disorders, as suggested by findings on IgE and antibody to *H. pylori *plasma levels [13,14], and by preliminary studies on overweight and insulin-resistant adults [21,22].

The collected evidence supports the interpretation that trained subjects learned to recognize the unconditioned feelings of hunger. The training in this study was intended to cut off excess food consumption, i.e. caloric intake occurring at high (> 85 mg/dL) glycemic concentrations, through conscious exposure of the subjects to the initial sensations of hunger arising when glycemia declined below 85 mg/dL. A 7-week period with association of estimated and measured glycemic values repeated 3 times per day was sufficient to train the subjects to accurately recognize their glycemia (estimation error < 3–5%; compared to 10–20% of controls; Table 2 and Figure 5), as tested at the final laboratory session. In addition to greater accuracy in glycemia estimation in the trained subjects compared to the control group, the data also indicate that estimated glycemic values were more accurate at glycemic concentrations below 87 mg/dL in both groups, independently of the feelings of hunger, compared to values estimated at high glycemic concentrations (Figure 5). Greater accuracy in the recognition of the sensations of initial hunger identified at low glycemic concentrations suggests that such feelings could be used as a reliable signal for meal consumption.

It is unlikely that the training per se simply established a new conditioning of the feeding behavior at lower glucose levels. First, most patients had pre-prandial high glycemic levels at baseline and they reported the sensations of initial hunger associated with low glycemia during the initial training as 'novel'. Second, during the first 3–4 days of training, the chosen sensations of initial hunger used to start a meal arose spontaneously (i.e. they were not triggered by external events related to food consumption, such as the sight of the dinner-table) and unexpectedly during working or entertaining activities, and persisted for at least 1 h. Third, at the final laboratory session, the number of trained subjects that recognized the appearance of sensations of initial hunger similar to those experienced during the training at low glycemic concentrations was significantly lower than in the control group (18 out of 64 *vs *42 of 72, respectively; Table 3) and the number of trained subjects who refused breakfast was significantly higher (39.1%) than that of controls (18.1%; Table 3). These observations suggest that the expression of a spontaneous and novel sensation of initial hunger at low glycemia in trained subjects did not simply reflect habitual repetition of a new conditioning caused by the training but rather was a cognitive ability to distinguish between low and high glycemic levels.

In previous studies, expressions of hunger have been reported at levels below about 60 mg/dL obtained following infusion of insulin or following prolonged food abstention [19,23]. These values are lower than those reported here. However, other studies showed that hunger in time-blinded subjects was preceded by transient blood glucose declines beginning at about 80 mg/dL [3-6,24] or, in some cases, at values as high as 100 mg/dL [5,24]. These data suggest that many factors – such as composition of previous meal, health status, age, and time-conditioning – affect the initiation of hunger and/or that different sensations of hunger are caused by separate mechanisms.

The arousal of unconditioned sensations of initial hunger below 87 mg/dL of glycemia in the trained subjects of our study is similar to the data of Melanson et al. [3] on time-blinded subjects in the morning who expressed hunger around 80 mg/dL after different lag times. In time-blinded young adults, Chapelot et al. [24] also showed that hunger expressions were associated with transient blood glucose declines from a mean glycemia of 100 mg/dL, but the subjects were conditioned by the past habit of snacking in the afternoon. The present study suggests that, in addition to time-blinding [3,24] and transient declines in blood glucose [[3,24], and this study], recognition of the sensations experienced during early training in association with low glycemia, as observed at the final session, indicate identification of unconditioned mechanisms of hunger in coincidence with metabolic insufficiency, as shown in rats by Nicolaiidis and Even [25]. This ability appeared particularly well trained in six subjects who reported that their hunger feelings outdoors, in the cold winter climate, faded indoors, in the overheated hospital rooms, due to decreased metabolic rate at high environmental temperature [26].

One possible explanation for the effects of training observed in this study is that, below a given level of glycemia, the trained subject responded to sensations of hunger similar to those that stimulate a two-year old child to demand food [11]. Young humans [24] and mice [6] accustomed to scheduled eating express hunger and transient blood glucose declines at a mean glycemia of 100 mg/dL. This implies that adults acquire conditioned hunger reflexes at high glycemia, leading to consumption of food in excess to what is necessary for energy balance. Consistent with this hypothesis is the observation that feeding 2-year-old children only following their unconditioned request of food succeeded in energy balance and body growth, and that their growth was associated with decreased energy intake [11-13] and with decreased metabolic rate [27].

Further data are necessary to establish if the training presented in this study will lead to reduced food intake and improvement of symptoms associated with gastro-intestinal disorders or other pathologic states [13,14,21,22].

## Conclusion

Humans can learn to distinguish the feelings of unconditioned hunger that arise at glycemic concentrations below 80–90 mg/dL from those that are conditioned and arise at glycemic concentrations higher than 80–90 mg/dL. This cognition may help in reducing conditioned eating in order to maintain energy balance.

## Abbreviations

BMI: body mass index, weight (kg)/square height (m)

r: linear correlation coefficient

## Competing Interests

The author(s) declare that they have no competing interests.
